# A Data-Driven Model with Feedback Calibration Embedded Blood Pressure Estimator Using Reflective Photoplethysmography

**DOI:** 10.3390/s22051873

**Published:** 2022-02-27

**Authors:** Jia-Wei Chen, Hsin-Kai Huang, Yu-Ting Fang, Yen-Ting Lin, Shih-Zhang Li, Bo-Wei Chen, Yu-Chun Lo, Po-Chuan Chen, Ching-Fu Wang, You-Yin Chen

**Affiliations:** 1 Department of Biomedical Engineering, National Yang Ming Chiao Tung University, Taipei 11221, Taiwan; newton1010.be04@nycu.edu.tw (J.-W.C.); crosshine@fda.gov.tw (Y.-T.F.); kk761003@nycu.edu.tw (S.-Z.L.); asd121212666.y@nycu.edu.tw (B.-W.C.); 2 Department of Cardiology, Ten-Chan General Hospital (Chung Li), Taoyuan 32043, Taiwan; skh0422@tcmg.com.tw; 3 Food and Drug Administration, Ministry of Health and Welfare, Taipei 11561, Taiwan; 4 Department of Internal Medicine, Taoyuan General Hospital, Ministry of Health and Welfare, Taoyuan 33004, Taiwan; lantin008@mail.tygh.gov.tw; 5 The Ph.D. Program for Neural Regenerative Medicine, Taipei Medical University, Taipei 11031, Taiwan; aricalo@tmu.edu.tw; 6 School of Electrical and Computer Engineering, Georgia Institute of Technology, Atlanta, GA 30332, USA; pchen353@gatech.edu; 7 Biomedical Engineering Research and Development Center, National Yang Ming Chiao Tung University, Taipei 11221, Taiwan

**Keywords:** blood pressure, photoplethysmography, machine learning, wearable devices, Gaussian process regression

## Abstract

Ambulatory blood pressure (BP) monitoring (ABPM) is vital for screening cardiovascular activity. The American College of Cardiology/American Heart Association guideline for the prevention, detection, evaluation, and management of BP in adults recommends measuring BP outside the office setting using daytime ABPM. The recommendation to use night–day BP measurements to confirm hypertension is consistent with the recommendation of several other guidelines. In recent studies, ABPM was used to measure BP at regular intervals, and it reduces the effect of the environment on BP. Out-of-office measurements are highly recommended by almost all hypertension organizations. However, traditional ABPM devices based on the oscillometric technique usually interrupt sleep. For all-day ABPM purposes, a photoplethysmography (PPG)-based wrist-type device has been developed as a convenient tool. This optical, noninvasive device estimates BP using morphological characteristics from PPG waveforms. As measurement can be affected by multiple variables, calibration is necessary to ensure that the calculated BP values are accurate. However, few studies focused on adaptive calibration. A novel adaptive calibration model, which is data-driven and embedded in a wearable device, was proposed. The features from a 15 s PPG waveform and personal information were input for estimation of BP values and our data-driven calibration model. The model had a feedback calibration process using the exponential Gaussian process regression method to calibrate BP values and avoid inter- and intra-subject variability, ensuring accuracy in long-term ABPM. The estimation error of BP (ΔBP = actual BP—estimated BP) of systolic BP was −0.1776 ± 4.7361 mmHg; ≤15 mmHg, 99.225%, and of diastolic BP was −0.3846 ± 6.3688 mmHg; ≤15 mmHg, 98.191%. The success rate was improved, and the results corresponded to the Association for the Advancement of Medical Instrumentation standard and British Hypertension Society Grading criteria for medical regulation. Using machine learning with a feedback calibration model could be used to assess ABPM for clinical purposes.

## 1. Introduction

Cardiovascular diseases (CVDs), a group of heart and blood vessel disorders, are the leading cause of death globally. Nearly 18 million people die of CVD annually, which is approximately one-third of all deaths in the world [1,2]. CVDs include cerebrovascular disease, coronary heart disease, congenital heart disease, and other conditions [3]. Recently, the American Heart Association (AHA) estimated that the global economic burden of non-communicable diseases related to CVDs was set to increase from USD 555 billion in 2015 to USD 1.1 trillion in 2035 [4]. Evidently, CVDs are already a serious health problem that should be solved and prevented.

Hypertension is a crucial cardiovascular parameter for the early identification of CVDs [5]. A key characteristic of CVDs is their dynamic self-regulation in the cardiovascular system, which involves multiple feedback control loops in response to variations in blood pressure (BP). Hence, continuous measurement of BP is necessary for medical diagnosis by physicians. A sphygmomanometer is a standard medical device that is used to monitor BP in the clinical setting. Although traditional 24-h BP measuring devices can monitor BP with a cuff at regular intervals through repeated inflation, such readings provide a tendency evaluation of the patient’s BP, which may not reflect the patient’s true BP. Additionally, measurement at night causes insomnia in healthy people, leading to increased awakenings. Such cuff-based methods are uncomfortable, discontinuous, and unsuitable for daily use. However, ambulatory BP monitoring (ABPM) without a cuff can be used to continuously detect rhythmic changes, which helps reduce the probability of false readings and understand the dynamic variability of BP [6]. ABPM is better than traditional BP measuring devices for real-time BP monitoring and could help in the prevention of CVD. Recently, wearable healthcare devices have been demonstrated to be successful for personal health monitoring over the long term and to help professionals understand how a patient’s multiple chronic conditions interact. To calculate BP using a wrist-type device, many studies have proposed the noninvasive solution of pulse transit time (PTT), which measures the time latency between the R-wave of electrocardiogram (ECG) and the peak of photoplethysmography (PPG) propagated from the heart to the wrist. PTT is the most common biomarker related to arterial elasticity and can be used to estimate systolic BP (SBP) and diastolic BP (DBP) [7]. Many studies have used a regression model to propose a relationship between BP and PTT [8,9]. This method has become widely recognized as a low-cost, non-invasive method for effectively estimating BP, which has been published in previous studies [10,11]. Some studies added personal information parameters, including the user’s height, weight, arm length, and morphological characteristics, as additional parameters for elevating the accuracy of BP estimation [12,13,14,15,16]. However, there is still another limitation regarding recording wrist-type ECG and PPG signals simultaneously, such as the minimum requirement of at least two electrodes that should be connected to both the right arm and left arm for standard lead I recording of the ECG signal. This results in the failure of the clinical application of ABPM. The PPT-based BP monitor does not function when the patient is asleep, and it is challenging to track abnormal variations in BP. Moreover, the electrode placed on the skin for long-term recording would cause irritation and degrade the ECG signal quality [17].

Some studies attempted to estimate BP using only PPG signals on the wrist-type device for simpler design and more convenient user scenarios without ECG electrodes [18,19,20,21,22]. The advantages are that irritation problems could be decreased and degradation in ECG signal quality could be avoided. A major benefit of using PPG-based BP estimation techniques is the continuous tracking of BP during daily routine activities and sleep, especially ABPM in clinical practice [23]. Additionally, the American Heart Association/American College of Cardiology (AHA/ACC) guideline indicated that out-of-office BP and home BP might be strongly correlated with cardiovascular mortality and morbidity compared with clinical BP [24], so the PPG-based BP estimation design provided a more suitable design for daily use. However, it seemed that the PPG-based BP estimation promised sufficient accuracy when the quality of the PPG signal was high. Many studies adopted features, such as temporal domain characteristics, including systolic time, diastolic time, cardiac period, and pulse width, by detecting various feature points of PPG signals for BP estimation [25,26]. Other PPG studies used frequency domain characteristics that contained valuable health-related information to estimate BP values based on Fast Fourier Transform and generalized transfer function [27,28]. In these studies of temporal and frequency domains, different machine learning (ML) algorithms were employed for BP estimation, such as regression algorithms [29], artificial neural networks (ANN) [30], fuzzy logic [31], and support vector machine [32]. Although the accuracy of these studies was high, too many parameters would result in massive memory usage for the model and a long computation time. It would not be appropriate to be implemented into the embedded system for wearable devices.

We compared PPG studies to estimate BP values. Some of these methods extended signal to several dimensions, which will enlarge the memory and inference time. The simple parametric models may lack expressive power, and another more complex method (such as neural networks) may not be easy to implement for real-time study. The advent of kernel machines, such as Support Vector Machines, classification trees, and Gaussian Processes [33], has allowed flexible models that are practical to work with microcontrollers. In regression ML models, it can automatically adapt to linear and nonlinear systems without the prior introduction of kernel functions. These methods have been applied in time series analysis, image processing, and automatic control. GPR models are an appropriate method to provide a probabilistic output based on the convenience properties of Gaussian processes and their kernel functions. Due to the Gaussian-based kernels and the normality of BP dataset distribution, it was more suitable to model the subject-specific relation between PPG and BP. The GPR model can also provide effective memory usage for the model and short computation time, so it is more suitable for BP estimation in embedded system designs for wearable devices. To assess the reliability and effectiveness of a PPG measurement method, it is essential to validate clinical data under serval experimental conditions. Usually, patients with hypertension also have other individual differences, which results in interference of PPG waveforms and decreases the fitting accuracy of the model. These individual differences can be affected by several factors, such as diabetes, arrhythmia, pregnancy, lifestyle, age, gender, body mass index, daily variations, or environmental conditions such as temperature and experimental errors during the data measurement [34,35,36,37,38]. As cardiovascular features and signals vary among conditions and change with time, determining how much the BP estimation varies depending on a factor or in terms of time is important.

The compensation of the human organ interaction and unexpected physiological variation and the time and environmental effect is a complex, ongoing problem. Therefore, inter- and intra-subject variability are the most important and traditional factors that cannot be personalized [39,40]. Such non-stationary interferences reduce performance and time and are only adaptable to a specific population, especially outside of the medical regulation standard [41]. Many studies have proposed strategies, such as the limitation for specific group application [42], short period [43], the use of multiple physiological and non-physiological parameters from different devices [44], and calibration with the standard device [45]. In this study, we reported that BP estimation based on multi-age-grouping models by PPG morphology characteristic parameters and personal information parameters as features could be a feasible method, but the calibration step is required. Calibration based on a linear method using a time delay and specific calibration intervals was proposed to maintain favorable accuracy, as reported previously [46]. Therefore, careful consideration is warranted for the selection of clinical populations to realize the calibration method so that the optimal accuracy and stability of readings can be ensured. A data-driven model with a feedback calibration method with a sphygmomanometer was proposed to solve the problem of the inter- and intra-subject variability.

An advanced solution was proposed in this study, and the details are as follows. First, a large PPG database for BP estimation could be analyzed from clinical studies, and the data were collected by the proposed reflective PPG sensor for the wearable device. This hardware mechanism of the sensor is simulated by optics and designed to reduce noise from motion artifacts (MAs) and ambient light. The PPG morphological characteristic parameter, personal information parameter, and actual BP values were explored and understood based on this database. Although many previous studies explored and defined the features of the waveform, there were still differences based on the hardware design, wearable method, and clinical indication [20,21,47]. A study highlighted the characterization of age-related changes in BP in normotensive and untreated hypertensive participants and showed that the pulsatile component of BP varies with age [48]. Therefore, before conducting data mining, grouping similar data by age range is important for exploratory participants. Age was demonstrated to be related to the hemodynamic model [49]. The grouping method is a common technique for statistical data analysis and is used in many fields, such as ML, data compression, pattern recognition, clinical trials, and computer graphics. The participants in the same group were more similar to each other than to those in other groups. Before constructing the model, a preprocess method can be used to reduce the complexity of training and increase the prediction accuracy.

In this study, all the participants were grouped into 11 sets belonging to different age groups. Then, the ML model was implemented as a predictor to extract the strong relative characteristics and estimate BP. However, the most accurate models of all groups were selected to implement in the wearable device. The ML model had some advantages of lower memory usage than the ANN model and higher accuracy than the multiple regression model. Based on an intelligent wearable biosensor design, the ML model was evaluated as the optimized model for the embedded system in the study. Moreover, personal information parameters, such as age, were found to be beneficial for reducing the inter- and intra-subject variability of PPG-based BP estimation for different pulse pressure (PP) ranges. Three other personal information parameters, such as body mass index, height, and weight, were not considered because of their temporary interference with personal behavior. The other parameters, such as gender and R-R interval, were used as features together with other PPG morphological characteristic parameters for the model input. Then, the adaptive calibration method was designed to enhance BP estimation, which could be increasingly accurate after learning patterns with actual BP input from the same person. By grouping the participants in the preprocessing stage, the variability could be subtracted to a small scale and the fitting errors could be significantly reduced, which could be better improved by a larger dataset with personal calibration. In short, to achieve the clinical application of the PPG-based BP estimation model, 24-h ABPM should be considered. This study proposed the data-driven model with feedback calibration implemented in the embedded system. The construction of the PPG database included different BP values and ages for multi-group ML models, and online learning by calibration using the sphygmomanometer was demonstrated to ensure the accuracy of BP estimation by avoiding inter- and intra-subject variability. Most importantly, clinical validation and regulatory considerations must be followed to guarantee functionality and efficiency before clinical application. Therefore, the clinical trial was designed to validate the results with and without calibration in a stable situation. The clinical trials representing the static accuracy of BP tried to effectively meet accuracy criteria of the Association for the Advancement of Medical Instrumentation (AAMI) standard and British Hypertension Society (BHS) guideline, and the results of a previous study [50,51]. The primary purpose of this study was to develop an intelligent wearable biosensor for BP monitoring based on the data-driven model. By using feedback calibration, inter- and intra-subject variability could be avoided, which was promising for long-term ABPM even during sleep.

## 2. Materials and Methods

### 2.1. A Proof-of-Concept Wrist-Type PPG Device for BP Estimation Using Reflective PPG

**Figure 1:**
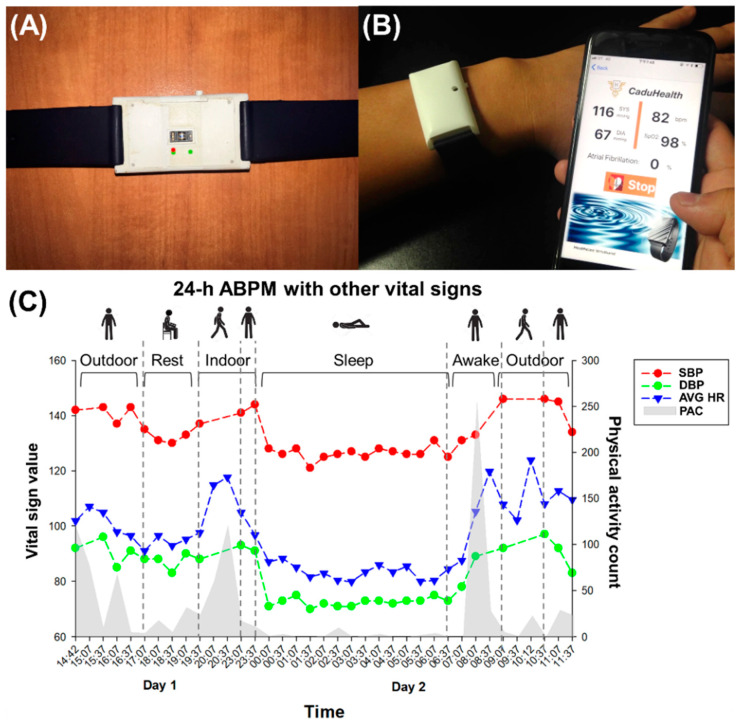
The proposed wrist-type PPG device for blood pressure estimation using reflective PPG signal. (**A**) The bottom of the proposed wrist-type PPG device integrated with a PPG biosensor that should be contacted with the skin inseparably. (**B**) In a practical wearing situation, the device was attached to the user’s wrist to enable the online measurements of PPG-based BP and other vital signs. The physiological data were wirelessly transmitted and shown in the mobile APP. (**C**) The APP also provided daily recording history of SBP, DBP, average HR (AVG HR), and physical activity count (PAC).

This study proposed a convenient wearable device for long-term monitoring of patients and healthy individuals. For a more convenient user scenario for tracking BP variation over 24-h, the all-day auto-monitoring mode was defined and implemented in the wrist-type PPG device. The PPG sensor was embedded in the back of the proof-of-concept device and was composed of a multi-wavelength light source with discrete green, red, and infrared light-emitting diodes (LEDs) and a photodiode (PD), as shown in Figure 1A. There was a black partition plate between the LED and PD, which could efficiently eliminate interference and crosstalk from external light [52]. After fastening the device on the wrist using a 20-mm wide silicone strap, the measured PPG-based BP and heart rate (HR), as well as oxygen saturation, could be transmitted wirelessly to a mobile phone for display and recording of multimodal physiological data in the mobile application (APP), as shown in Figure 1B. In this study, we focused on BP estimation, and we further presented an ML framework based on the wearable device to deal with raw PPG data. Furthermore, the APP possesses an auto-monitoring mode to perform measurements every 30 min based on the stable user scenario during daytime and nighttime for the clinical use of 24-h ABPM. [53,54,55]. Figure 1C showed an example for a BP trend with different sampling data points (i.e., high sampling in sleep or rest state and low sampling in high activity count state). This study implemented the motion detection method with a 3-axis accelerator in wearable devices as the preprocessing stage for steady-state measurement, and users in the iOS application recorded the participant’s posture by themselves. For example, if this participant was sitting with low physical activity counts from 17:07 to 19:37 on day 1, the BP estimation values were stable and periodic recorded during rest time. Instead, the BP estimation was not available because of the motion in an upright position from 19:37 to 23:07 on day 1 and from 9:07 to 10:37 on day 2. Moreover, the mean BP in the supine position was lower than in the sitting and upright positions. In summary, the 24-h BP variability using the proposed wearable device was affected by physical activity and transitions between postures (i.e., supine, sitting, and upright) consistency with the traditional ABPM [56].

**Figure 2:**
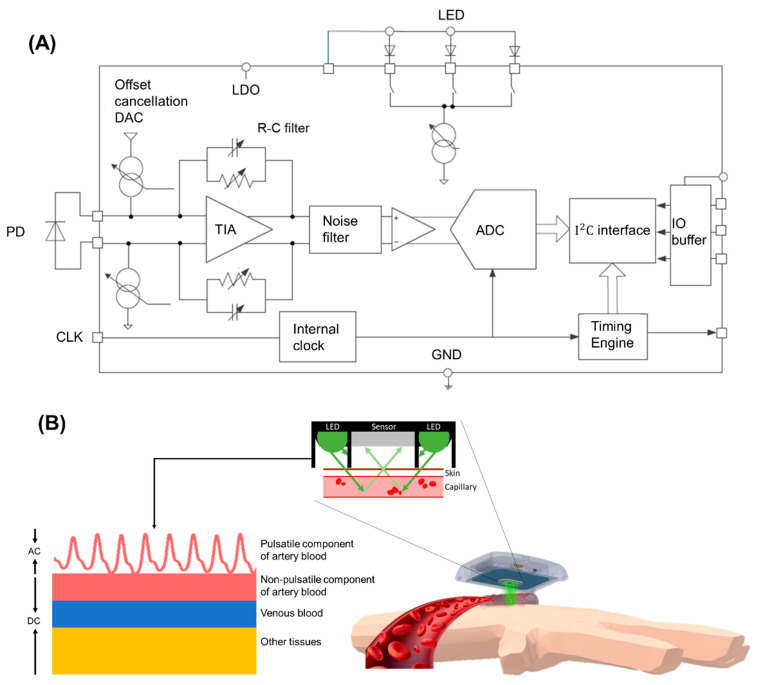
(**A**) Shows the circuit of the PPG analog front end. It contained an LED, adjustable R-C filter, TIA to enhance the signal from the PD and convert the signal to digital data by ADC. (**B**) The optical principle of the PPG sensor that reflecting the blood volume variation from PPG signal. The PPG sensor with the optical shielding design for decreasing noise and disturbance acquired reflective PPG signal including AC and DC components. AC was defined as the pulsatile component of the artery and DC was consisted of non-pulsatile components of artery blood, venous blood, and other tissues.

The on-board microcontroller unit (MCU) is an ARM^®^ Cortex^®^-M4 core with Bluetooth Low Energy (BLE) module (BMD-300-A-R, Rigado Inc., Portland, OR, USA), running with a maximum working frequency in 64 MHz, and also equipped with 512 KB of flash and 64 KB of RAM memory. Additionally, the device consisted of a PPG sensor (IMSA805, ITM Semiconductor Co., Ltd., Cheongju-si, Korea) used to continuously measure the PPG signal in pulse width modulation, and a 3-axis acceleration (MC3610, mCube Inc., San Jose, CA, USA) used to reflect the MA were co-implemented in the adaptive noise cancellation from measured PPG signal [57] and used to record participant’s physical activities [58] as well. The power consumption of the device was 15.58 mW, which allowed continuous recording up to 15 h or measurements at 30-min intervals for up to 6 days with an 80-mAh battery. The device’s available RAM (64 KB) was not enough to store signals and model parameters and perform the computation. The raw PPG data were not stored in an embedded system, and BP values were transmitted to the APP. For this reason, 62 KB of RAM was used for computing variables and firmware code. The 276 KB of flash memory was used for storing model parameters (i.e., Exponential GPR model). In this study, only the green light LED was turned on to measure BP. The green light was transmitted by an LED into the skin, and the amount of reflective or unabsorbed light was measured using a PD, which showed the blood volume changes in the microvascular bed of the tissue. The back-scattered or reflected PPG signal detected from the PD was first amplified (gain = 66 dB) and low-pass filtered (filter bandwidth = 50 Hz) in the analog front-end, which utilized a trans-impedance amplifier (TIA) with the direct current (DC) cancellation loop and a band pass filter to compensate for the DC drift, as shown in Figure 2A. The gain of TIA was set by its feedback resistor $\left(R_{f}\right)$ and could be set from 10 kΩ to 2 MΩ. The TIA gain between the input current and output differential voltage of the TIA was equal to $2R_{f}$. At the output of the TIA was a switched resistor-capacitor $\left(R_{f},C_{f}\right)$ low-pass analog filter for rejecting common-mode noise and noise related to power supplies. The effective bandwidth of the switched resistor-capacitor filter was approximately 50 Hz. After the analog signal was preprocessed, it was transferred to a digital signal by a 24-bit analog-to-digital converter (ADC). Then, the band-pass digital filter was used at 0.5–10 Hz, which filtered the noise signal, and it comprised the highest frequency cutoff, which was higher than the frequency of the clean PPG signal, and the lowest frequency cutoff, which was lower than the frequency of breathing and exercise (15–20/min). Therefore, the clean PPG signal was obtained and used for the pulse characteristic calculation.

As shown in Figure 2B, the PPG signals acquired from the PPG sensing module could be individually divided into the DC and alternating current (AC) components. The DC component was attributed to the bulk absorption of the skin, muscle, venous blood, non-pulsatile components of artery blood, and unfluctuating tissue. The AC component was directly attributable to a pulsatile component of arterial blood and showed the changes in the blood volume that occurred between the systolic and diastolic phases of the cardiac cycle. The green light was used to observe the PPG waveform because of the large AC component and good signal-to-noise ratio. Each cardiac cycle was determined as a pressure pulse wave when the blood was perfused to the dermis and subcutaneous tissue of the skin in each ventricular contraction.

In this study, The PPG method could be used to directly measure the blood flow pulse on the skin, and the wrist-type PPG device could analyze the vital signs uploaded from the BLE communication protocol. The BP values could be displayed on the cell phone with the ML model. Moreover, the ML model in the device could be updated online by adding actual BP values to yield more accurate results.

### 2.2. Clinical Trial for Validation of the Difference of Estimated and Actual BP

The clinical protocol was approved by the institutional review board (IRB) of National Yang-Ming University (IRB No. YM106096E) and the IRB of Taoyuan General Hospital (IRB No. TYGH104055). In total, 435 participants (male: female = 244:191; mean age, 38.498 years; age range, 15–94 years) were included in this study. The acceptance criteria for the participants are indicated in Table 1.

**Figure 3:**
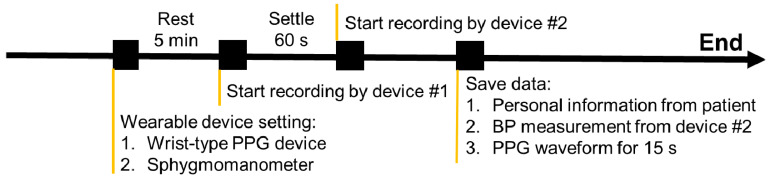
The experimental protocol for the validation of the BP estimation based on wrist-type PPG device. The protocol was designed for static state validation. Note that the resting for 5 min should be conducted at the beginning of the experimental protocol to reduce the interference of the environment and physiological variation. The protocol was consisting of the 5 min rest state and 60-s settle state for simultaneously recording with wrist-type PPG device and sphygmomanometer.

The clinical trial was designed for the static state validation, as indicated in Figure 3. This study collected actual BP and 15-s PPG signals in series during the data collection process. The participants were asked to rest for 5 min before the start of recording, and their health status was assured. Using the wrist-type PPG device, which was proposed in this study, pulsatile PPG data of every participant was recorded for at least 1 min at 256 Hz using a triaxial accelerometer. Every participant was instructed to breathe as normal, sit, and remain stable during the data collection. Then, reliable BP data were labeled using an FDA-approved sphygmomanometer for BP monitoring (JPN-700, Omron Corporation, Kyoto, Japan) by a clinical technician and clinical cardiologist. The dataset included actual BP and PPG data and information from participants who were diagnosed with normotension (BP < 130/90 mm Hg), prehypertension, and hypertension by two specialists. The personal information of the participants was recorded for detailed analysis and classification. All data, including vital signs from the wearable devices and personal information, were recorded for the following analysis.

### 2.3. ML-Based BP Estimation with the Calibrated Model by Age Grouping

**Figure 4:**
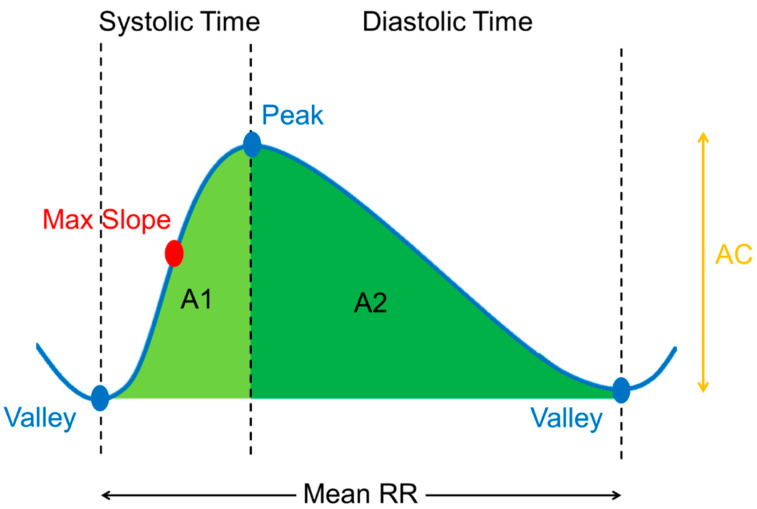
The characteristics of PPG morphology corresponds to the pulsatile PPG waveform including waveform parameter and time-related parameter. Waveform parameter: systolic area over total area, diastolic area over total area, systolic area over pulse amplitude, diastolic area over pulse amplitude, maximal amplitude over time as maximal slope. Time-related parameter: systolic time, diastolic time and mean peak to peak interval.

Referring to past research [59,60], the PPG wristband, which received the CE certificate of conformity, was used for short-term measurement and signal analysis. By evaluating the 24-h signal quality, the 15-s measurement time was considered the optimal sampling time for physiological applications of PPG [59]. Error reduction with averaging for PPG estimation was reported in another study [60]. In this study, the error between actual and estimated BP was calculated after 15-s pulse averaging and corresponded to the medical regulatory error boundary as defined by the AAMI, which was 5 ± 8 mmHg. Therefore, the green light PPG morphological characteristics could be defined as the mean of the 15-s segment pulsatile PPG of the systolic area over the total area, diastolic area over the total area, systolic area over the pulse amplitude, diastolic area over the pulse amplitude, maximal amplitude over time, systolic time, diastolic time, and mean PP interval, as shown in Figure 4. Personal information parameters, such as age and gender, were added for the BP prediction model. Age and gender are important factors when considering phenotypic changes in health and disease. It was demonstrated that the blood volume of the cardiac cycle from a vessel is significantly impacted by gender and age [61]. The estimated results were evaluated for age groups (5–50 years) and without grouping. The participants were considered to be normally distributed after grouping. Table 2 shows the characteristic parameters of the PPG waveform and their corresponding personal information parameters for the BP prediction model and calibration model. Moreover, the actual BP of the user was defined as a factor to calibrate and optimize the BP model.

The variation of PPG morphology in characteristics was significantly different between males and females and different age groups. In order to reduce the variation, multiple models were constructed for subject-specific relation between PPG and BP. The models included multiple groups by age grouping method trained using exponential GPR algorithm. The partitions of the training set and testing set were seven and three, so the PPG database was divided into training dataset (306 participants as reference database) and test dataset (129 participants as validation database). Ten variables including PPG morphology characteristic parameters and personal information parameters were the model input parameters.

Following the details of GPR was reported previously in [33], the training set was expressed as Equation (1).

(1) $${\left\{x_{i},\;y_{i}\right\}}_{i=1}^{n}$$

where n represents the number of data sets, x represents the training parameters array including PPG morphology characteristic parameter and personal information parameter as shown in Table 2, and y represents the target value as actual BP value. A learning function $f\left(x_{i}\right)$ was used for transforming the input array $x_{i}$ into the target value $y_{i}$ given a model as Equation (2).

(2) $$y_{i}=f\left(x_{i}\right)+\varepsilon_{i}$$

where $\varepsilon_{i}$ represents Gaussian noise with zero mean and $\sigma_{n}^{2}$ represented the variance. As a result, the observed targets can also be described by a Gaussian distribution as Equation (3).

(3) $$y\;\sim \;N\left(0,K\left(x,\;x\right)+\sigma_{n}^{2}I\right)$$

where x represents the vector of all input points $x_{i}$ and K(x, x) the covariance matrix computed using a given covariance function. The covariance function could be defined by various kernel functions and could be parameterized in terms of the kernel parameters in vector ***θ***. Hence, it was possible to express the covariance function as K(x, x|θ). This model used the exponential kernel function with a separate length scale for each predictor. The covariance function was defined as follows:

(4) $$k\left(x_{i},\;x_{j}|\theta \right)=\sigma^{2}{}_{f}\;exp\left[-\frac{\sqrt{{\left(x_{i}-x_{j}\right)}^{T}\left(x_{i}-x_{j}\right)}}{\sigma_{l}}\right]$$

The kernel parameters were based on the signal standard deviation $\sigma_{f}$ and the characteristic length scale $\sigma_{l}$. The unconstrained parametrization θ was:

(5) $$\theta_{1}=log\sigma_{l},\theta_{2}=log\sigma_{f}$$

Therefore, the joint distribution of the observed target values and predicted value $f\left(x_{i}\right)$ for a query point i was given in Equation (6).

(6) $$\left[\begin{array}{c} y\\ f\left(x_{i}\right) \end{array}\right]\;\sim \;N\left(0,\left[\begin{array}{cc} K\left(x,\;x\right)+\sigma_{n}^{2}I & k\left(x,\;x_{i}\right)\\ k\left(x_{i},\;x\right) & k\left(x_{i},x_{i}\right) \end{array}\right]\right)$$

The predicted mean value $\bar{f\left(x_{i}\right)}$ and the corresponding variance $V\left(x_{i}\right)$ could be represented in Equations (7) and (8) as follows:

(7) $$\bar{f\left(x_{i}\right)}=k{\left(x,\;x_{i}\right)}^{T}{\left(\;K\left(x,\;x\right)+\sigma_{n}^{2}I\right)}^{-1}y$$

(8) $$V\left(x_{i}\right)=k\left(x_{i},x_{i}\right)-k{\left(x,\;x_{i}\right)}^{T}{\left(\;K\left(x,\;x\right)+\sigma_{n}^{2}I\right)}^{-1}k\left(x,\;x_{i}\right)\;$$

The GPR model is a type of ML method for statistically analyzing data. The purpose is to understand the relationship between two or more variables and establish a mathematical model to predict the variables of interest. More specifically, using regression analysis, the relation function can be found and the long-term trend of BP can be estimated from the given PPG characteristic.

**Figure 5:**
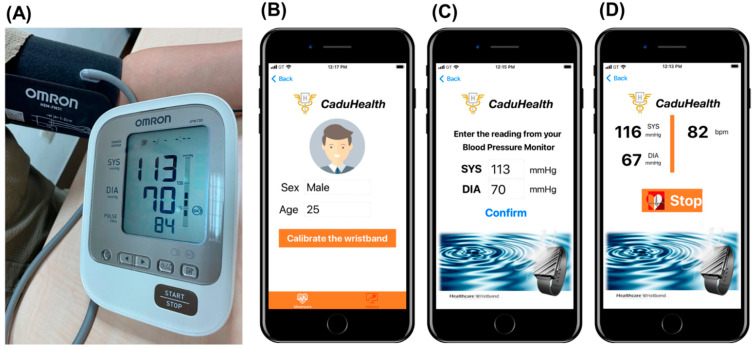
The details about proposed feedback calibration embedded in PPG devices. (**A**) The calibration system. (**B**) Step 1. Setting Up. (**C**) Step 2. Calibration. (**D**) Step 3. Measuring Blood Pressure.

The detail of the BP calibration procedure in our study was given as below and shown in Figure 5:

The calibration system was shown in Figure 5A.

Step 1. Setting Up: Before you start measuring your blood pressure, you must use APP to connect to the wearable device, and set up your personal profile (Age, Gender) in Figure 5B.

Step 2. Calibration: To ensure more accurate measurements, be sure to calibrate your wrist with an upper-arm, cuff-based blood pressure monitor. Start the blood pressure measurement on the cuff-based blood pressure monitor and enter the reading in the APP before BP estimation in Figure 5C.

Step 3. Measuring Blood Pressure: Perform the measurement stable and sitting in a quiet place. The wrist will start measurement every 15 s automatically. The BP estimation result will be saved in the APP in Figure 5D.

**Figure 6:**
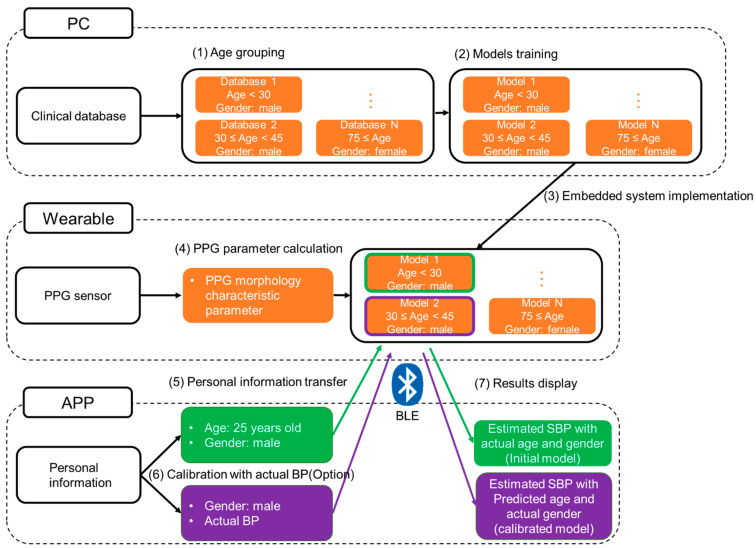
The details of the block diagram of the calibration were divided into seven steps (i.e., (1) Age grouping; (2) Models training; (3) Embedded system implementation; (4) PPG parameter calculation; (5) Personal information transfer; (6) Calibration with actual BP; (7) Results display).

In Figure 6, the details of the block diagram of the comparison process were divided into seven steps. All PPG data from our clinical database were first grouped by age and trained as the different models individually. For DBP model in this study, the PPG database was separated by gender and age by groups of 15 years (i.e., Age < 30, 30 ≤ Age < 45, 45 ≤ Age < 60, 60 ≤ Age < 75, 75 ≤ Age). Above all, there are 10 groups trained as different models for SBP. On the other hand, the SBP model was by groups of 30 years with 6 groups (i.e., Age < 30, 30 ≤ Age < 60, 60 ≤ Age). Then, these models were all implemented in our proposed embedded system. When a participant (i.e., 25 years old) started active BP estimation without calibration, all the age groups of trained-based models with the actual gender (i.e., male) were used to predict many BP values. The minimal mean error between the predicted SBP (i.e., 143 mmHg) and actual SBP (i.e., 140 mmHg) from cuff-based blood pressure monitor (JPN-700, Omron Healthcare Co. Ltd., Terado-cho, Japan) was calculated and the corresponded optimal age group (i.e., 30 ≤ Age < 45) was selected. Finally, the optimal age group for this participant was used for the further BP estimation accurately. In order to demonstrate the practical application, the application interface based on the iOS app was designed as the easily keying in the personal information parameters and activating calibrated function by with Bluetooth Low Energy (BLE) in our wearable device. The chosen model was more suitable for the participant’s PPG parameters, and a compensated value could be calculated in variable $k\left(x_{i},x_{i}\right)$ and BP could be predicted using Equation (7) initially. Then, the parameters could be included in the model for personal calibration, and the optimized model would predict the BP more accurately.

### 2.4. Statistical Analysis of BP Estimation in Accordance with International Standards

The BP data of all participants were examined for normality statistics called the Shapiro–Wilk (S–W) test and the results before and after the age grouping were compared [62]. The S–W test had been shown to be capable of detecting normality for a wide variety of statistical distributions. The *p*-value of the S–W method tested the normality of the data. The higher *p*-value did not reject the null hypothesis which conformed to the normal distribution, and the lower *p*-value represented that rejecting the null hypothesis which does not conform to the normal distribution.

To evaluate the best grouping-year-number with age by groups of different years, Pearson correlation analysis was conducted to identify age without grouping (100 years) and with grouping. Furthermore, the regression analysis presented the correlation results for performance comparison of different algorithms. These algorithms included 20 ML models. Significant correlations (*p* < 0.05) were reported.

To evaluate the accuracy of the proposed wrist-type PPG device, two international protocols of BHS guideline and AAMI standard were considered. Both standards defined a maximum-tolerated error between BP monitoring by the proposed wrist-type PPG device and FDA-approved electric sphygmomanometer in the static state. The standard accuracy criteria were defined and described as follows. The BHS grading criteria were the cumulative percentage in 5, 10, and 15 mmHg, with four grades: Grade A (≤5, 60%; ≤10, 85%; and ≤15, 95%), Grade B (≤5, 50%; ≤10, 75%; and ≤15, 90%), Grade C (≤5, 40%; ≤10, 65%; and ≤15, 85%) and Grade D (worse than Grade C). Alternatively, AAMI was used to conduct a statistical comparison as the estimation error of BP (ΔBP = actual BP—estimated BP) with standard deviation (≤5 ± 8 mmHg). A zero ΔBP presented the accurate model, whereas its negative and positive values would indicate BP overestimation and underestimation, respectively. Meanwhile, the Pearson correlation analysis and Bland–Altman analysis were the visualized approaches for statistical evaluation of agreement between estimated BP value and actual BP value.

## 3. Results

### 3.1. Evaluation of Best Performing ML-Based Algorithm for BP Estimation

**Figure 7:**
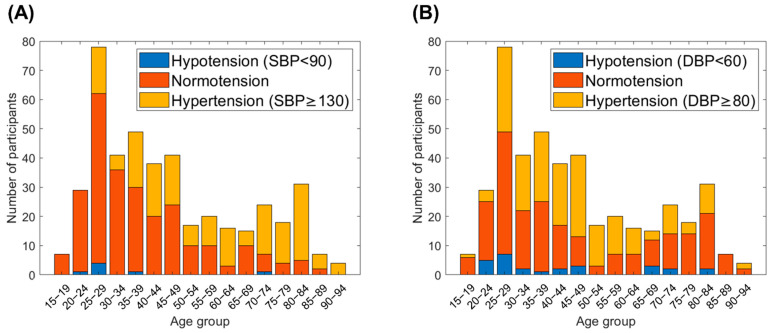
The distribution of actual SBP and DBP levels over different age groups in our clinical database. (**A**) Distribution of SBP levels. (**B**) Distribution of DBP levels. Blue column represents the number of participants in hypotension. Orange column represents the number of participants in normotension. Yellow column represents the number of participants in hypertension. The age of participants covered from 15 to 94 years old. The hypertension and the hypotension populations were also included which was suitable for the true population.

In this study, the PPG database included 435 participants with 15-s pulsatile PPG signals. The recorded data could be divided into three BP levels (i.e., normotension, hypertension, and hypotension) based on their actual BP values. The normal BP was within the range of <130/80 mmHg in SBP/DBP and ≥ 90/60 mmHg in SBP/DBP. The abnormal BP levels were defined as hypertension (SBP/DBP ≥ 130/80 mmHg) and hypotension (SBP/DBP < 90/60 mmHg), either, according to the guidelines of ACC/AHA and National Health Service (NHS). A stacked histogram was used to plot the age group corresponding to the number of participants in the actual SBP and DBP (Figure 7A,B, respectively). The age distribution is as follows: 15–19 years, 20–24 years, … 90–94 years. It is clear that most participants were aged 25–29 years, and most participants had normotension. The percentage and number of participants with hypertension increased gradually with age increasing. The age distribution of all participants in the clinical database reflected that of the true population.

As mentioned, extracted characteristics were considered input parameters for ML regression algorithms. The models were trained using the training set, and BP was estimated using testing data in each of the twenty ML methods. Table 2 indicates the proposed 10 PPG features in the BP estimator. Table 3 shows a comparison of the accuracy between the ML models for SBP and DBP, including the ΔBP and its standard deviation of the estimated BP values and actual BP values. The best ML model was selected as a proposed estimator for BP value if it contained the minimum ΔBP among all results. Thus, the exponential GPR method was the optimal ML model with the lowest ΔBP (SBP: −0.7167 ± 15.5851, DBP: −0.8693 ± 12.6172). However, all the results did not meet the international standard for medical regulations, with Grade D showing results of 5, 10, and 15 mmHg.

**Figure 8:**
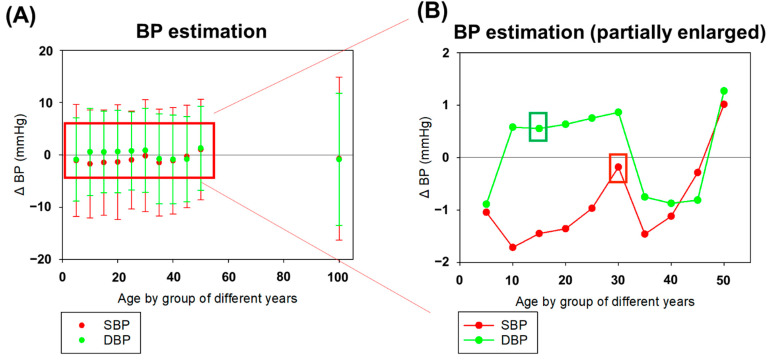
The estimation error of BP (ΔBP) with various periods of grouping years (5–50 years) and without grouping (100 years) in the exponential GPR model. (**A**) The ΔBP and its standard deviation in SBP and DBP. The red line represented SBP results and the green line represented DBP results. (**B**) The partial enlarged detail of ΔBPs of SBP and DBP for grouping the period of 5, 10, 15, 20, 25, 30, 35, 40, 45, 50 years. We found the period of 30 years for grouping had the lowest ΔBP in SBP and the 15-year grouping had the lowest ΔBP in DBP. All correlation results were similar with statistical significance (*p* < 0.001).

Figure 8A shows the 11 different periods of age groups in the exponential GPR model. The age without grouping (100 years) had a high level of ΔBP and standard deviation among all the results. After grouping the participants by age, the ΔBP and the standard deviation reduced. As shown in Figure 8B, the age group of 15 years had the lowest ΔBP and standard deviation in DBP, which was 0.5539 ± 7.8138 mmHg. The age group of 30 years had the lowest ΔBP in SBP, which was −0.1809 ± 10.7177 mmHg. Thus, the best estimation of SBP and DBP was in different age groups, in which DBP was much smaller than SBP. The correlation results between different grouping-year-number were evaluated in Figure 8. The correlation coefficient *r*-value and *p*-value were calculated to evaluate the optimal grouping-year-number as statistical indexes. In the results, optimal grouping-year-number was 30 years in SBP (*r* = 0.538, *p* < 0.001) and 15 years in DBP (*r* = 0.373, *p* < 0.001). These *r*-values were similar between with age grouping and without age grouping, but all the correlation results were statistically significant (*p* < 0.001).

**Figure 9:**
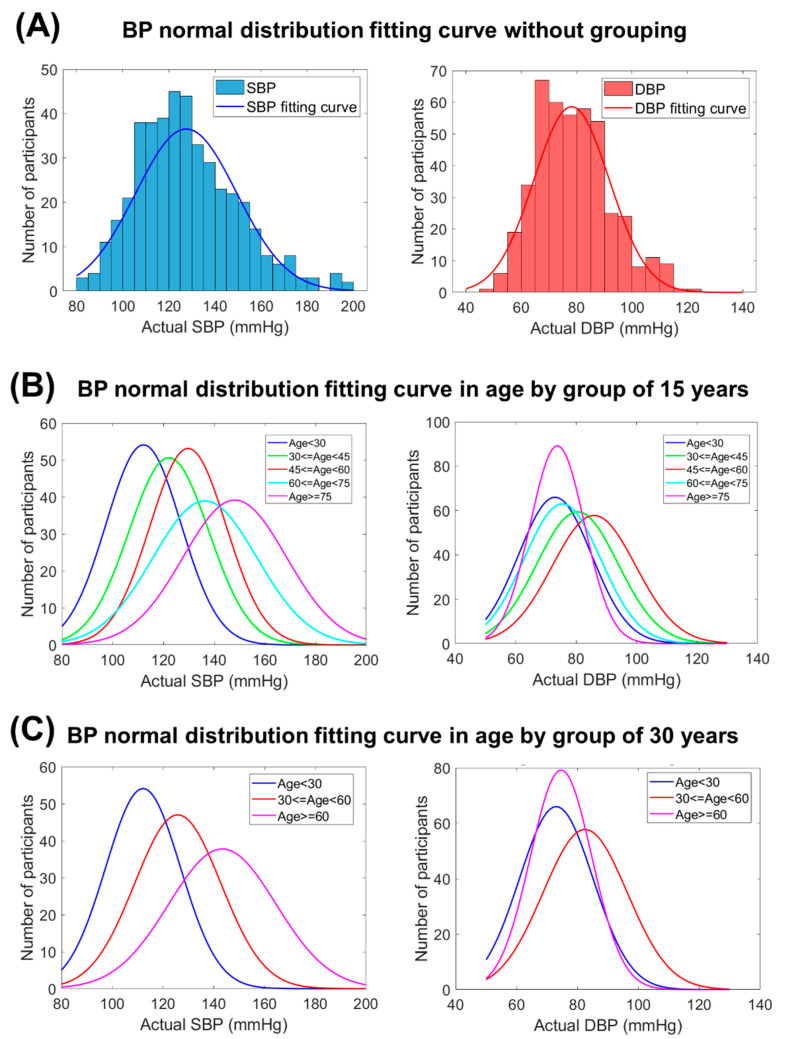
BP distribution with grouping and without grouping. (**A**) BP normal distribution fitting curve without grouping. (**B**) BP normal distribution fitting curve in age by group of 15 years. (**C**) BP normal distribution fitting curve in age by group of 30 years. The distribution fitting curves used to depict distribution normality. After grouping participants, these curves were closer to the null hypothesis of standard normal distribution test which was more suitable for exponential GPR training model. In Figure 9B,C, the fitting curves of actual SBP and DBP shifted to higher-value along with older age groups. Nevertheless, the fitting curves of actual DBP shifted back to lower-value when the age was older than 60 years (light blue and purple line).

In Figure 9, the distribution and the fitting curves were used to depict distribution normality with grouping and without grouping in this study. With grouping participants, the fitting curves of actual SBP and DBP shifted to higher-value along with older age groups. Nevertheless, the fitting curves of actual DBP shifted back to lower-value when the age was older than 60 years. Using the Shapiro–Wilk normality test, the *p*-value (*p* = 1.6 × 10^−10^ for SBP and *p* = 2.29 × 10^−11^ for DBP in Figure 9A) was found to be very small without grouping, which represented the non-normal distribution characteristic. As shown in Figure 9B, the normal distribution fitting curve for the age group of 15 years had the following *p*-values (*p* = 3.59 × 10^−5^, 1.24 × 10^−4^, 1.63 × 10^−5^, 9.69 × 10^−3^, and 2.05 × 10^−3^ for SBP and *p* = 7.74 × 10^−7^, 3.16 × 10^−4^, 1.41 × 10^−2^, 5.04 × 10^−2^, and 3.52 × 10^−3^ for DBP with five age groups: <30 years, 30–45 years, 45–60 years, 60–75 years, and ≤75 years). As shown in Figure 9C, the normal distribution fitting curve for the age group of 30 years had the following *p*-values (*p* = 3.59 × 10^−5^, 6.91 × 10^−7^, and 9.85 × 10^−5^ for SBP and *p* = 7.74 × 10^−7^, 2.91 × 10^−5^, and 1.91 × 10^−4^ for DBP with three age groups: <30 years, 30–60 years, and ≤60 years). All the *p*-values were closer to the null hypothesis of standard normal distribution, which was more suitable for the exponential GPR training model.

### 3.2. Comparison of the Proposed BP Estimation Model with and without Calibration

**Figure 10:**
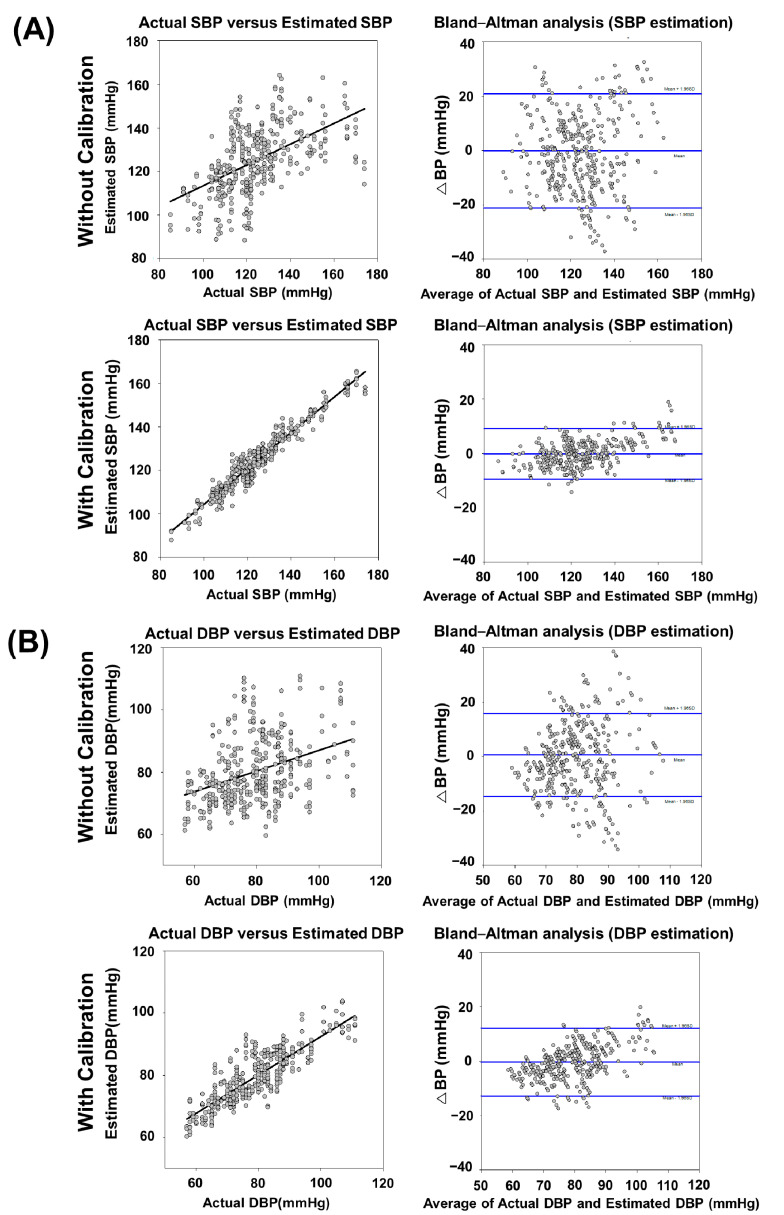
Relationship between actual BP measurement and exponential-GPR estimated BP in the analysis of correlation coefficient and Bland-Altman plot for ΔBP between actual BP measurement and exponential-GPR BP estimation. (**A**) SBP measurement without calibration showed a moderate correlation (*r*-value = 0.538 and its associated *p* = 1.84 × 10^−30^) with a mean ΔBP of −0.1809 mmHg (CI = −21.1876 to 20.8258) between actual BP measurement and exponential-GPR BP estimation. SBP measurement with calibration showed an excellent correlation (*r*-value = 0.968 and its associated *p* = 1.20 × 10^−232^) with a mean ΔBP of −0.1776 mmHg (CI = −9.4603 to 9.1051) between actual BP measurement and exponential-GPR BP estimation. (**B**) DBP measurement without calibration showed a fair degree of relationship (*r*-value = 0.373 and its associated *p* = 3.10 × 10^−15^) with a mean ΔBP of 0.5539 mmHg (CI = −14.7611 to 15.8689) between actual BP measurement and exponential-GPR BP estimation. DBP measurement with calibration showed an excellent correlation (*r*-value = 0.854 and its associated *p* = 3.81 × 10^−111^) with a mean ΔBP of −0.3846 mmHg (CI = −12.8674 to 12.0982) between actual BP measurement and exponential-GPR BP estimation. The mean ΔBP of the data was illustrated by the central horizontal line (blue) of Bland–Altman analysis. Upper and lower reference lines (blue) showed the upper and lower limits of agreement (95% confidence intervals (CIs)).

Figure 10 indicated comparisons of correlation and Bland–Altman analysis for BP validation between without and with calibration. In the correlation analysis, the correlation coefficient *r*-value and *p*-value were 0.538 (*p* = 1.84 × 10^−30^) and 0.373 (*p* = 3.10 × 10^−15^) in SBP and DBP, respectively. With calibration, the *r*-value and *p*-value were 0.968 (*p* = 1.20 × 10^−232^) and 0.854 (*p* = 3.81 × 10^−111^) which means the increasing of correlation between actual BP and estimated BP. Similarly, the Bland–Altman plots showed that the mean value of SBP and DBP were similar between without and with calibration, and the standard deviation was much smaller after calibration in both SBP and DBP results. The performance comparison of exponential GPR-estimated BPs between with and without calibration was shown in Table 4. The results in Table 4 were expressed in (1) Total Mode and (2) Interval Mode, which both were from BP estimated data of the same study group (129 testing participants) for further performance analysis. In Total Mode, corresponding summary (combined) BP estimated results were determined from the overall 129 testing participants in this study. In the Interval Mode, the corresponding BP estimated results were individually classified into three BP subgroups of normotension, hypertension, and hypotension from 129 testing participants. In Total Mode, as shown in the upper row of Table 4, the cumulative percentage of readings within 15 mmHg was 70.974% (Grade D) in SBP and 78.072% (Grade D) in DBP. The ΔBP of SBP was −0.1809 ± 10.7177 mmHg and of DBP was 0.5539 ± 7.8138 mmHg. The results from the proposed prediction model did not correspond to the medical regulatory error boundary as defined by the AAMI, which is 5 ± 8 mmHg. For the calibration result, the same testing participants were used by using actual BP values to optimize the models. The cumulative percentage of readings within 15 mmHg improved to 99.225% (Grade A) in SBP and 98.191% (Grade A) in DBP. The ΔBP of SBP was −0.1776 ± 4.7361 mmHg and of DBP was −0.3846 ± 6.3688 mmHg. The calibration results from the proposed estimation model corresponded to the medical regulatory error boundary defined by the AAMI very well. In Interval Mode, as shown in the lower row of Table 4, the SBP and DBP estimation using the exponential GPR method with calibration also achieved an overall B/A grading and fulfilled the AAMI/ BHS accuracy criteria [51,63]. Overall, the exponential GPR-BP estimation with calibration showed higher accuracy performances than those without calibration in both Total and Interval Modes.

## 4. Discussion

Focusing on wearable devices and their healthcare purpose, this BP measurement method was based on simplifying the data collection steps and reducing the number of steps. However, a wrist-type PPG device was selected without any controlling steps. Suppose the accuracy of estimation was sufficiently enhanced with calibration, a cuffless BP meter that predicts ABPM instead of using cuff-based BP devices can be implemented in the future. Most vital signals in clinical settings are continuously measured, except BP. However, physicians are also concerned about potential cardiovascular diseases based on patients’ vital signs, especially daily BP trends. To provide a warning signal, trends in BP values can be obtained when training the model with real data in actual clinical settings.

A wrist-type device of photoplethysmography was provided as a monitoring tool for healthcare to estimate BP and examine the use of PPG independently in this study. The widespread use of BP estimation tools in the health care domain was indicated, and the increasing number of PPG-based wearable devices was also given with continuous ambulatory measurements of BP. In most cases, traditional readings were taken every 30- to 60-min intermittent sampling of the recordings during the day and night when the patient was in a stable situation [64]. This previous study also highlighted that the performance of noninvasively functioning ambulatory monitors tended to be poorer under ambulatory conditions, in the working environment, and during exercise than at rest. Therefore, the criteria were applicable only to subjects examined at rest. In view of the above-mentioned, the wearable BP estimation in this study was designed under the same condition (i.e., at rest) and took the readings only at 15-s intervals. In order to get the high quality of data in both traditional and wearable PPG methods, the limitation for keeping the arm steady during measurement was required.

Figure 7 shows a histogram of the age distribution of the participants from 15 to 94 years from the PPG database with low, normal, and high SBP and DBP. The BP distribution of all the participants was in accordance with the definition of the general population in the ISO 81060-2:2018 standard. According to the BP distribution pattern, in the database, at least 5% of the readings should have a reference SBP of ≤100 mmHg, and at least 5% of the readings should have a reference SBP of ≥160 mmHg. The distribution of BP was compliant with the standard.

Table 3 shows the comparison results of the model for training and evaluating the BP estimation values, and exponential GPR ML algorithms performed the final model with the highest accuracy. Due to the nonlinear relationship between BP and the PPG signals, the linear regression algorithm did not explain which ΔBP was highest and the exponential GPR method with the lowest ΔBP and its standard deviation. Compared with the ML methods, the exponential GPR method results in the database in this study produced results that were similar to the AAMI standard and BHS standard. Based on the results, the exponential GPR method based on green light wrist-type PPG features could be a powerful method for BP prediction.

As shown in Figure 8, the ΔBP result of grouping the participants according to age in the exponential GPR model was more accurate than with no grouping. Normally, SBP is 90–200 mmHg and DBP is 60–130 mmHg. As the ranges of SBP and DBP are different, SBP and the DBP had different age intervals in the training results. The age interval was smaller in DBP because of the narrower range. Otherwise, the age interval in SBP was larger because of the wide range in SBP distribution. As shown in Figure 9, the higher age range led to a wider distribution in the BP histogram before the grouping operation. Using the Shapiro–Wilk normality test, the *p*-value was found to be much lower than 0.05 in all data, which represented the non-normal distribution characteristics. To increase the suitability of the model for exponential GPR training, using the grouping operation by age interval was a common preprocess method before model construction to reduce the complexity of training and increase the predicted accuracy. After the grouping operation, the *p*-value increased, which indicated the reason why grouping by age before the estimation would increase the accuracy. The best model in all age ranges can be selected for use in personal calibration.

As shown in Table 4 and Figure 10, after grouping the data, the results showed that the accuracy was increased without calibration but was still not high enough for the standard of the FDA-approved sphygmomanometer. We suspected that the data after grouping contained intra-/inter-individual variability. The physiological status of peripheral blood vessels, such as stiffness, compliance, and aging, was generally accepted, which can be partially expressed in terms of peripheral arterial pressure waveforms. The BP varied in the same age groups, even though the corresponding physiological parameters of PPG were similar. As the chronological age, which is the number of years a person had been alive, was well known and not the same as physiological age, it was necessary to determine the physiological age. Nonetheless, the phenomenon of BP variation over time could still be observed. Using the personal calibration method, accurate BP values were measured, and the success rate of the personal calibration method was increased. The major core concept of the calibration method in our study was different from a previous study [29]. In the Bland–Altman analysis for the BP estimation by the exponential GPR model with the calibration, as shown in Figure 10, significant BP prediction biases of underestimations (positive ΔBPs) and overestimations (negative ΔBPs) were found in these subgroups of the Interval Mode (Table 4). The inference was mainly that the under-representation associated with imbalanced source data from relatively small numbers of participants with hypertension and hypotension in our training set, which led to the biased PPG-based BP estimation in the hypertensive and hypotensive BP ranges [65]. In the Total Mode, as shown in Table 4, the nearly zero mean of ΔBP showed from a statistical perspective in the lump-sum condition (a whole distribution of BP testing data) that it would diminish their corresponding biases of underestimation and overestimation in the BP predictions of three subgroups of the Interval Mode and could, therefore, be used in calibration with the traditional cuff-based BP measurement to provide accurate estimations of continuous BP monitoring. However, we considered that only one-point calibration was not enough to refine the general model successfully, especially for long-term monitoring. More individual data points were needed as the refined training process to maintain accuracy. Instead, the age grouping method was proposed in this study as multiple models from different age groups. Our core concept of the calibration method was to fit the best model among all age groups as the optimal individual model. It could solve the problem of the lack of insufficient individual reference points. The results corresponded to the medical regulatory error boundary defined by the AAMI. This enabled the realization of a data-driven model with feedback calibration-embedded BP estimator using reflective photoplethysmography, which can be potentially used in ABPM in clinical settings.

## 5. Conclusions

In this study, a new approach for BP estimation was proposed, which was continuous, noninvasive, and based on using only the PPG signal. The method proposed was based on the nonlinear ML GPR model, which could estimate the regression between BP values and PPG features by grouping the age range of a user. The results demonstrate the potential of the proposed ML model for ABPM. According to our estimated results after calibration, the conditions were matched to the AAMI standard requirements. The ΔBP was negligible, and standard deviation was close to the standard AAMI limit in SBP estimation. This achievement was due to the large variety of PPG parameters, grouping the set-in different age ranges, and calibration by the standard used in the implementation of the ML method design. The ML algorithms in BP estimation achieved grades A and B according to the BHS standard. Calibration methods could be implemented in an embedded system for personalized measurement and be adapted to different environments and health statuses. With the use of all-day auto-monitoring, sufficient feasible data could be obtained in models for further expert application by self-training and population learning. Above all, the proposed system could be linked to medical and fitness applications and potentially extend to other domain applications such as insurance and nutrition.

## Figures and Tables

**Table 1:** The details of clinical trial database collection for BP estimation modeling.

Criterion	Details
Age	≥18 years (males and females).
Document	Willing to voluntarily sign the study-specific informed consent form.
History	No previous percutaneous coronary intervention, coronary artery bypass graft, abdominal aortic aneurysm, peripheral vascular disease, aortic stenosis, arrhythmia, tremors (before or during procedure), diabetes, kidney disease, or carotid bruits.
Clinical trial setting	SBP ranged from 80 mmHg to 250 mmHg and DBP ranged from 40 mmHg to 150 mmHg.
	In a controlled laboratory environment, with constant temperature, pressure, and silence ensured.

**Table 2:** PPG morphological characteristic parameters and personal information parameters for the BP prediction model.

Item	PPG Morphological Characteristic Parameter		Personal Information Parameter	
	PPG Waveform Parameter	PPG Time-Related Parameter (Unit: s)		
Without Calibration	A1/(A1 + A2) A2/(A1 + A2) A1/AC A2/AC Max Slope	Systolic Time Diastolic Time Mean RR	Real	
			Age (y/o) Gender (0 or 1)	
With Calibration	A1/(A1 + A2) A2/(A1 + A2) A1/AC A2/AC Max Slope	Systolic Time Diastolic Time Mean RR	Real (For initial use)	Optimized
			SBP (mmHg) DBP (mmHg)	Age (y/o) Gender (0 or 1)

**Table 3:** Comparison of accuracy between all ML models for SBP and DBP without calibration and age grouping.

Blood Pressure	SBP					DBP				
Model	*r*	≤5 (%)	≤10 (%)	≤15 (%)	ΔBP (mmHg)	*r*	≤5 (%)	≤10 (%)	≤15 (%)	ΔBP (mmHg)
Exponential GPR (this study)	0.44 ***	27.131 (D)	56.072 (D)	67.441 (D)	−0.716 ± 15.5851	0.31 ***	35.401 (D)	55.814 (D)	77.261 (D)	−0.869 ± 12.6172
Bagged Trees	0.43 ***	32.041 (D)	53.746 (D)	71.576 (D)	3.141 ± 14.9823	0.23 ***	32.041 (D)	58.914 (D)	77.002 (D)	1.846 ± 12.2447
Boosted Trees	0.43 ***	25.581 (D)	50.387 (D)	67.441 (D)	1.145 ± 16.1572	0.28 ***	30.491 (D)	55.038 (D)	77.519 (D)	−1.328 ± 12.4168
Coarse Gaussian SVM	0.40 ***	20.930 (D)	44.702 (D)	57.622 (D)	−3.328 ± 19.2503	0.25 ***	34.366 (D)	60.465 (D)	77.519 (D)	−1.964 ± 12.4512
Coarse Tree	0.37 ***	25.581 (D)	50.387 (D)	70.801 (D)	−1.402 ± 15.0477	0.18 ***	35.142 (D)	64.082 (D)	81.657 (D)	1.782 ± 11.6775
Cubic SVM	0.32 ***	22.222 (D)	43.152 (D)	62.015 (D)	−1.943 ± 18.1104	0.21 ***	28.423 (D)	50.129 (D)	72.35 4(D)	−1.015 ± 13.7282
Fine Gaussian SVM	0.30 ***	20.413 (D)	46.511 (D)	66.149 (D)	−2.324 ± 16.3242	0.24 ***	30.232 (D)	51.938 (D)	73.901 (D)	−0.801 ± 14.0499
Fine Tree	0.28 ***	23.772 (D)	45.219 (D)	68.733 (D)	−1.861 ± 16.5967	0.21 ***	33.333 (D)	59.173 (D)	77.261 (D)	−1.869 ± 12.6172
Interactions Linear	0.39 ***	17.312 (D)	40.051 (D)	58.656 (D)	−5.003 ± 17.5311	0.28 ***	19.638 (D)	42.118 (D)	64.599 (D)	−1.835 ± 16.0832
Linear	0.31 ***	23.772 (D)	50.646 (D)	68.992 (D)	1.746 ± 20.0023	0.18 ***	33.850 (D)	61.757 (D)	78.553 (D)	1.508 ± 12.0335
Linear SVM	0.32 ***	23.255 (D)	47.803 (D)	66.667 (D)	−1.068 ± 16.0259	0.21 ***	32.558 (D)	60.981 (D)	79.586 (D)	1.221 ± 12.7356
Matern5/2 GPR	0.43 ***	24.031 (D)	51.938 (D)	71.317 (D)	1.345 ± 15.6983	0.30 ***	32.041 (D)	62.532 (D)	81.395 (D)	2.237± 12.549
Medium Gaussian SVM	0.47 ***	21.705 (D)	44.444 (D)	64.857 (D)	−1.943 ± 17.5536	0.30 ***	33.333 (D)	58.139 (D)	77.519 (D)	−1.877 ± 12.8238
Medium Tree	0.34 ***	22.739 (D)	41.860 (D)	63.307 (D)	−2.063 ± 18.2236	0.14 ***	25.839 (D)	46.253 (D)	69.251 (D)	−1.018 ± 14.9782
Quadratic SVM	0.44 ***	25.581 (D)	47.028 (D)	66.149 (D)	−1.875 ± 15.978	0.29 ***	36.692 (D)	61.498 (D)	78.294 (D)	−1.325 ± 11.6168
Rational Quadratic GPR	0.43 ***	20.413 (D)	41.860 (D)	60.465 (D)	−4.198 ± 17.2031	0.31 ***	32.816 (D)	59.431 (D)	77.002 (D)	−1.838 ± 12.7787
Robust Linear	0.32 ***	24.547 (D)	53.488 (D)	72.351 (D)	1.496 ± 15.1054	0.19 ***	32.816 (D)	60.465 (D)	79.586 (D)	1.279 ± 12.7838
Squared Exponential GPR	0.41 ***	24.806 (D)	45.736 (D)	63.824 (D)	−1.344 ± 15.9769	0.32 ***	32.041 (D)	58.139 (D)	76.227 (D)	−1.883 ± 12.9418
Stepwise Linear	0.40 ***	25.323 (D)	47.545 (D)	67.183 (D)	−1.587 ± 16.163	0.26 ***	33.333 (D)	60.206 (D)	79.069 (D)	1.587 ± 12.2077
Gaussian Mixture Model	0.17 **	4.333 (D)	11.333 (D)	16.676 (D)	−21.937± 38.1851	0.12 *	13.000 (D)	21.000 (D)	35.672 (D)	−17.211± 30.0149

BHS grading criteria (mmHg, cumulative percentage): Grade A (≤5, 60%; ≤10, 85%; and ≤15, 95%), Grade B (≤5, 50%; ≤10, 75%; and ≤15, 90%), Grade C (≤5, 40%; ≤10, 65%; and ≤15, 85%), Grade D (worse than Grade ***C***). ANSI/AAMI/ISO 81060-2:2013: ΔBP < 5-mmHg, mean standard deviation < 8-mmHg. *p*-value: * *p* < 0.05, ** *p* < 0.01, *** *p* < 0.001.

**Table 4:** Performance comparison of the BP estimation between without and with calibration.

Exponential GPR Model		Without Calibration				With Calibration			
Total Mode		≤5 (%)	≤10 (%)	≤15 (%)	ΔBP (mmHg)	≤5 (%)	≤10 (%)	≤15 (%)	ΔBP (mmHg)
DBP		37.936 (D)	63.637 (D)	78.072 (D)	0.5539 ± 7.8138	60.723 (A)	88.372 (A)	98.191 (A)	−0.3846 ± 6.3688
SBP		37.421 (D)	58.379 (D)	70.974 (D)	−0.1809 ± 10.7177	71.834 (A)	96.382 (A)	99.225 (A)	−0.1776 ± 4.7361
**Interval Mode**		**≤5 (%)**	**≤10 (%)**	**≤15 (%)**	**ΔBP (mmHg)**	**≤5 (%)**	**≤10 (%)**	**≤15 (%)**	**ΔBP (mmHg)**
DBP	hypotension <60	16.667 (D)	33.333 (D)	60.000 (D)	−12.5832 ± 5.5526	50.000 (B)	80.000 (B)	100.000 (A)	−7.5400 ± 3.7221
	normotension 60–79	39.891 (D)	65.295 (C)	78.689 (D)	−7.5627 ± 6.8504	65.027 (A)	89.617 (A)	97.814 (A)	−3.9673 ± 4.7367
	hypertension ≥80	29.101 (D)	58.201 (D)	77.249 (D)	6.5413 ± 9.9935	59.788 (B)	87.831 (A)	98.413 (A)	3.6523 ± 5.2502
SBP	hypotension <90	20.000 (D)	33.333 (D)	66.667 (D)	−11.1238 ± 3.6607	53.333 (B)	100.000 (A)	100.000 (A)	−5.5844 ± 2.3086
	normotension 90–129	40.621 (C)	61.136 (D)	72.348 (D)	−4.6248 ± 9.9551	73.863 (A)	98.482 (A)	100.000 (A)	−1.5600 ± 3.9808
	hypertension ≥130	20.000 (D)	42.500 (D)	56.667 (D)	8.1414 ± 12.3490	68.333 (A)	91.674 (A)	97.501 (A)	2.9987 ± 4.7528

BHS grading criteria (mmHg, cumulative percentage): Grade A (≤5, 60%; ≤10, 85%; and ≤15, 95%), Grade B (≤5, 50%; ≤10, 75%; and ≤15, 90%), Grade C (≤5, 40%; ≤10, 65%; and ≤15, 85%), Grade D (worse than Grade C). ANSI/AAMI/ISO 81060-2:2013: ΔBP < 5-mmHg, mean standard deviation <8-mmHg.

## Data Availability

The datasets generated for this study are available on request to the corresponding author.
